# Complete loss of the atrial natriuretic peptide‐converting enzyme Corin and CHAF‐LA syndrome: Implications to natriuretic peptide physiology and left atrium health

**DOI:** 10.1002/ctm2.1540

**Published:** 2024-01-15

**Authors:** Alina Kurolap, Chofit Chai Gadot, David Zahler, Jacob N Ablin, Hagit Baris Feldman

**Affiliations:** ^1^ The Genetics Institute and Genomics Center, Tel Aviv Sourasky Medical Center Tel Aviv Israel; ^2^ Faculty of Medicine Tel Aviv University Tel Aviv Israel; ^3^ Department of Cardiology Tel Aviv Sourasky Medical Center Tel Aviv Israel; ^4^ Internal Medicine H and Institute of Rheumatology, Tel Aviv Sourasky Medical Center Tel Aviv Israel

**Keywords:** ANP, atrial natriuretic peptide, atriopathy, Corin

1

The natriuretic peptide (NP) hormone system has been extensively studied in the context of blood pressure homeostasis and cardiovascular disease.^1^ Two main NPs ‐ atrial natriuretic peptide (ANP) and B‐type/ brain NP (BNP) ‐ are secreted from the left atrial and ventricular cardiomyocytes, respectively, following stimuli, such as fluid overload, sympathetic stimulation, and hypernatremia.^1^ ANP and BNP are synthesized in cardiomyocytes as preprohormones, where they are cleaved to produce prohormones. These prohormones – proANP and proBNP – undergo additional proteolytic cleavage by the transmembrane enzymes Corin and Furin to remove the N‐terminal region and form the mature and active hormones, which are released to the bloodstream (Figure 1).^1^ Interestingly, while proBNP can be cleaved by both Furin and Corin, proANP is cleaved exclusively by Corin.^1^ ANP and BNP exert their physiological responses through binding NPR‐A (also known as GC‐A) receptors and activating cGMP signaling in target organs (Figure 1), where they promote vasodilation (in blood vessels), natriuresis and diuresis (in the kidney), inhibit the renin‐angiotensin‐aldosterone pathway, and protect against hypertrophy, fibrosis and apoptosis (in the heart).^1^, ^2^ Due to their similar structure and overlapping functions, ANP and BNP are often discussed interchangeably in the literature.^1^

**FIGURE 1:**
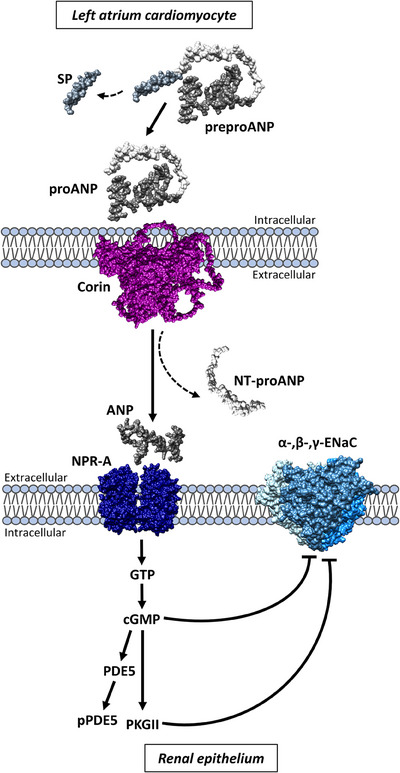
Atrial natriuretic peptide (ANP) processing and downstream pathway. An illustration of ANP synthesis and processing in cardiomyocytes, and its downstream pathway in the kidney. ProANP is cleaved by Corin to form mature ANP, which is released into the bloodstream. ANP binds its receptor (known as NPR‐A or GC‐A) in the kidneys and activates cGMP signaling. This leads to inhibition of the epithelial sodium channel (ENaC) and promotes natriuresis. SP = signal peptide.

Human monogenic diseases and animal models have long been explored to study the function and physiology of specific genes and proteins in vivo. Several genetic disorders involving the NP pathway have been described to date, providing the medical and scientific communities an overlook of how the disruption of each step in this pathway affects body function. For example, pathogenic variants in *NPPA* (encoding the ANP precursor protein, OMIM *108780) cause autosomal dominant (AD) atrial fibrillation (AFib) with dilated cardiomyopathy and fibrotic atrial myopathy, and autosomal recessive (AR) atrial standstill; bi‐allelic pathogenic *NPR1* variants (encoding the NPR‐A receptor, OMIM *108960) have recently been described in two families with neonatal systemic hypertension;^3^ and gain‐of‐function variants in genes encoding the ENaC subunits (*SCNN1A/B/G*, OMIM *600228, *600760, *600761) cause AD Liddle syndrome, characterized by hypertension, and AR pseudohypoaldosteronism, with cardiovascular involvement (hypotension, cardiac arrest and ventricular arrhythmia).

In our study,^4^ recently published in the *New England Journal of Medicine*, we described two siblings of Filipino descent who presented with a novel cardiovascular syndrome involving cardiomyopathy, hypertension, arrhythmia and fibrosis of the left atrium (CHAF‐LA) syndrome (Figure 2). This syndrome is caused by complete loss of the ANP‐converting enzyme Corin due to a homozygous loss‐of‐function variant in the *CORIN* gene (NM_006587.4: c.684dupG; p.Met229Aspfs*16)^4^; *CORIN* has been previously implicated in preeclampsia (OMIM *605236). The homozygous c.684dupG variant leads to complete loss of plasma Corin and the consequent absence of mature ANP (as inferred by negligible levels of plasma NT‐proANP); plasma BNP levels are elevated, most likely in compensation for the lack of ANP.^4^ This phenotype largely recapitulates the *Corin* knockout mouse model, first described 18 years before the human patients.^5^ Not only do these findings prove that indeed only Corin is able to cleave proANP,^1^ but they also provide an opportunity to isolate the physiological functions unique to ANP, that is, not shared with BNP. Although elevated, BNP could not completely stabilize the patients’ blood pressure, and patient plasma could not inhibit ENaC in vitro compared to healthy individuals.^4^ This suggests that both hormones are needed for homeostasis, and may reflect the role of renal Corin in ANP signaling, natriuresis and diuresis.^2^

**FIGURE 2:**
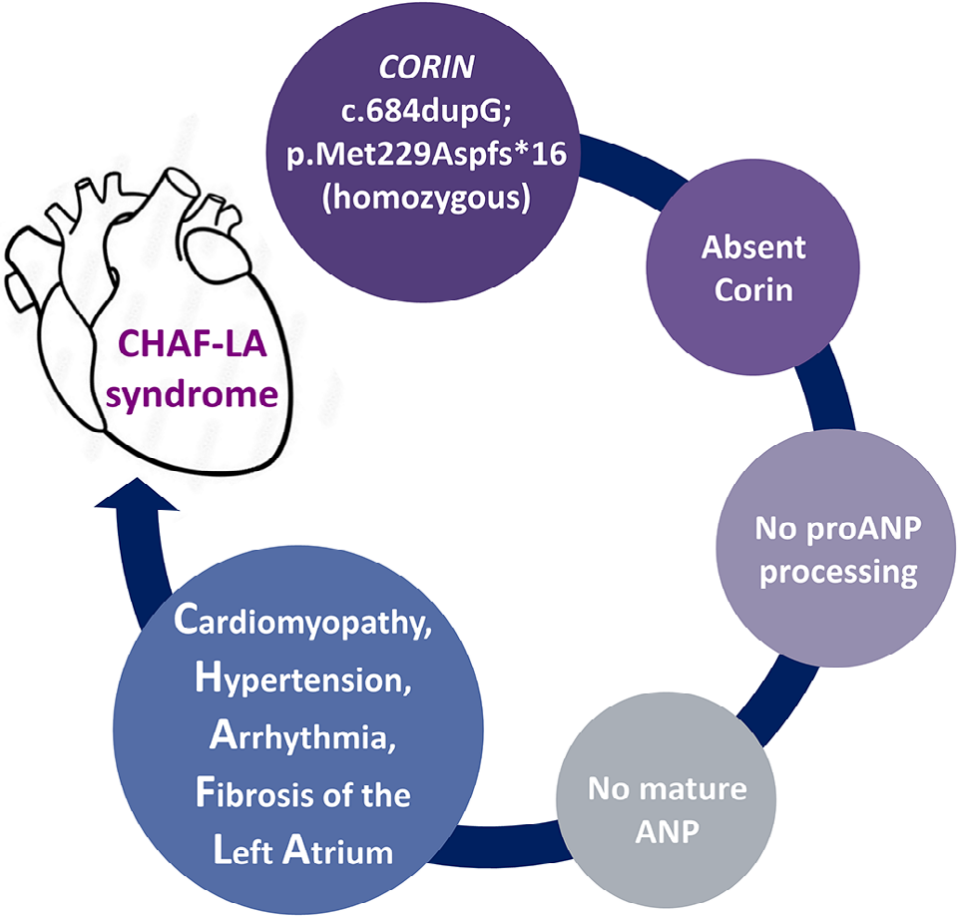
Corin loss and CHAF‐LA syndrome. A homozygous loss‐of‐function variant in the gene encoding the atrial natriuretic peptide (ANP)‐converting enzyme Corin leads to the absence of mature and active ANP and causes a disorder characterized by cardiomyopathy, hypertension, arrhythmia and fibrosis of the left atrium (CHAF‐LA syndrome).

Hypertension and cardiovascular disease are prevalent in the Philippines. Interestingly, a recent study found that a common belief among hypertensive Filipino patients is that the cause of their ailment lies in ‘nasa dugo’, meaning ‘it's in the blood’ ‐ or ‐ inherited.^6^ In line with this, the family history of the siblings with CHAF‐LA syndrome reveals multiple family members with early onset hypertension, which is also associated with a *CORIN* variant carrier status. The variant observed in the studied family has been observed only in East Asian individuals in gnomAD (“Southeast” category is not available) with an allele frequency of 0.1304% in this population (rs756399499), which may reflect a genetic risk factor for hypertension in the Philippines. An extended study exploring loss‐of‐function *CORIN* variants in healthy and hypertensive individuals is required to fully delineate the association and disease risk. Moreover, loss‐of‐function variant carriers presenting with cardiovascular disease should be methodically phenotyped to understand the full extent of their pathology, especially in comparison with the homozygous CHAF‐LA patients. This would be important for tailoring treatment, especially for those patients with drug‐resistant hypertension, as in the studied family.

AFib is highly associated with myocardial fibrosis and atrial tissue remodeling.^7^ Indeed, the main pathology in both patients is attributed to left atriopathy, manifesting as extensive fibrosis, persistent arrhythmia (AFib and flutter) and cardiomyopathy,^4^ likely due to the absence of the anti‐inflammatory, anti‐fibrotic and anti‐hypertrophic properties attributed to ANP in atrial cardiomyocytes.^2^ Interestingly, ANP/proANP is aggregation‐prone and tends to accumulate in the atria with age, a pathology known as isolated atrial amyloidosis.^8^ ANP‐positive amyloids are also observed in AFib patients.^8^ Since a subcategory of myocardial fibrosis relates to amyloid deposition, and the two cannot be differentiated using imaging, unprocessed proANP accumulation in the left atrium of Corin deficient patients could be the key etiology of the observed fibrosis.

Arrhythmic resistance is strongly associated with atrial fibrosis and amyloidosis.^7^ While catheter ablation has limited success in controlling amyloidosis‐related arrhythmia, systemic targeted treatment improved arrhythmic control in these patients.^9^ Furthermore, *Corin* overexpression in mice, leading to ANP hyper‐activation, reduced cardiac fibrosis.^10^ These findings suggest cardiovascular potential benefits for increasing ANP production in Corin deficient patients. Several NP‐based therapeutics are in development.^1^ Since native ANP has an extremely short half‐life, it requires a continuous intravenous infusion, which cannot be used in chronic patients. Therefore, MANP – a more stable modified ANP, has been developed. MANP has shown favorable antihypertensive, natriuretic and diuretic properties in animal models and in humans,^11^ and should be considered in resistant hypertension and AFib patients, focusing on those carrying *CORIN* loss‐of‐function variants and CHAF‐LA syndrome patients. Soluble Corin infusion transiently restored proANP cleavage in *Corin* knockout mice,^5^ and should also be explored further in human cardiovascular disease therapeutics.

## AUTHOR CONTRIBUTIONS

Not Applicable.

## CONFLICT OF INTEREST

Not Applicable.

## ETHICS STATEMENT

Not Applicable.

## References

[1] Goetze JP , Bruneau BG , Ramos HR , Ogawa T , de Bold MK , de Bold AJ . Cardiac natriuretic peptides. Nat Rev Cardiol [Internet] 2020;17(11):698‐717. Available from: doi: 10.1038/s41569-020-0381-0 32444692

[2] Bie P . Natriuretic peptides and normal body fluid regulation. Compr Physiol 2018;8(3):1211‐1249. 29978892 10.1002/cphy.c180002

[3] Capri Y , Kwon T , Boyer O , et al. Biallelic NPR1 loss of function variants are responsible for neonatal systemic hypertension. J Med Genet 2023;60(10):993‐998. 37080586 10.1136/jmg-2023-109176 PMC10579472

[4] Baris Feldman H , Chai Gadot C , Zahler D , et al. Corin and left atrial cardiomyopathy, hypertension, arrhythmia, and fibrosis. N Engl J Med 2023;389(18):1685‐1692. 37913506 10.1056/NEJMoa2301908

[5] Chan JCY , Knudson O , Wu F , Morser J , Dole WP , Wu Q . Hypertension in mice lacking the proatrial natriuretic peptide convertase Corin [Internet]. 2005 [cited 2020 Jun 22]. Available from: www.pnas.orgcgidoi10.1073pnas.0407234102 10.1073/pnas.0407234102 PMC545541 15637153

[6] Lasco G , Mendoza J , Renedo A , et al. Nasa dugo (It's in the blood’): Lay conceptions of hypertension in the Philippines. BMJ Glob Heal 2020;5(7):1‐8. 10.1136/bmjgh-2020-002295 PMC7351273 32646854

[7] Marrouche NF , Wilber D , Hindricks G , et al. Association of atrial tissue fibrosis identified by delayed enhancement MRI and atrial fibrillation catheter ablation: The DECAAF study. Jama 2014;311(5):498‐506. 24496537 10.1001/jama.2014.3

[8] Van Den Berg MP , Mulder BA , Klaassen SHC , et al. Heart failure with preserved ejection fraction, atrial fibrillation, and the role of senile amyloidosis. Eur Heart J 2019;40(16):1287‐1293. 30753432 10.1093/eurheartj/ehz057 PMC6553504

[9] Donnellan E , Wazni OM , Hanna M , et al. Atrial fibrillation in transthyretin cardiac amyloidosis: predictors, prevalence, and efficacy of rhythm control strategies. JACC Clin Electrophysiol 2020;6(9):1118‐1127. 32972546 10.1016/j.jacep.2020.04.019

[10] Sullivan RD , Houng AK , Gladysheva IP , et al. Corin overexpression reduces myocardial infarct size and modulates cardiomyocyte apoptotic cell death. Int J Mol Sci 2020;21(10):3456. 32422879 10.3390/ijms21103456 PMC7278931

[11] Cannone V , Burnett JC . Natriuretic peptides and blood pressure homeostasis: implications for MANP, a novel guanylyl cyclase a receptor activator for hypertension. Front Physiol 2022;12(February):1‐8. 10.3389/fphys.2021.815796 PMC8878907 35222065

